# *Nfib* hemizygous mice are protected from hyperoxic lung injury and death

**DOI:** 10.14814/phy2.13398

**Published:** 2017-08-22

**Authors:** Vasantha H. S. Kumar, Joseph Chaker El Khoury, Richard Gronostajski, Huamei Wang, Lori Nielsen, Rita M. Ryan

**Affiliations:** ^1^ Department of Pediatrics University at Buffalo Buffalo New York; ^2^ Department of Pediatrics Virginia Commonwealth University Richmond Virginia; ^3^ Departments of Biochemistry and Developmental Genomics & Genetics Genomics & Bioinformatics Program University at Buffalo Buffalo New York; ^4^ Department of Pediatrics Medical University of South Carolina Charleston South Carolina

**Keywords:** Death, hyperoxia, inflammation, lung injury, *Nfib* hemizygous mice

## Abstract

Nuclear Factor I (*Nfi)* genes encode transcription factors essential for the development of organ systems including the lung. *Nfib* null mice die at birth with immature lungs. *Nfib* hemizygous mice have reduced lung maturation with decreased survival. We therefore hypothesized that these mice would be more sensitive to lung injury and would have lower survival to hyperoxia. Adult *Nfib* hemizygous mice and their wild‐type (Wt) littermates were exposed to 100% O_2_ for 89, 80, 72 and 66 h for survival studies with lung outcome measurements at 66 h. *Nfib* hemizygous and Wt controls were also studied in RA at 66 h. Cell counts and cytokines were measured in bronchoalveolar lavage (BAL); lung sections examined by histopathology; lung angiogenic and oxidative stress gene expression assessed by real‐time PCR. Unexpectedly, *Nfib* hemizygous mice (0/14–0%) had significantly lower mortality compared to Wt mice (10/22–45%) at 80 h of hyperoxia (*P* < 0.003). LD_50_ was 80 h in the Wt group versus 89 h in the hemizygous group. There were no differences in BAL cell counts between the groups. Among the cytokines studied, MIP‐2 was significantly lower in hemizygous mice exposed to hyperoxia. New vessel formation, edema, congestion, and alveolar hemorrhage were noted on histopathology at 72 and 80 h in wild‐type mice. *Nfib* hemizygous lungs had significant downregulation of genes involved in redox signaling and inflammatory pathways. Adult *Nfib* hemizygous mice are relatively resistant to hyperoxia compared to wild‐type littermates. Mechanisms contributing to this resistance are not clear; however, transcription factors such as *Nfib* may regulate cell survival and play a role in modulating postnatal lung development.

## Introduction

Despite advances in neonatology, bronchopulmonary dysplasia (BPD) remains a major cause of morbidity and mortality in premature infants. The pathophysiology of BPD is multifactorial, with mechanical ventilation, inflammation, infection, hyperoxia, and genetics all playing a role in the development of BPD (Ryan et al. 2008). However, novel interventions to reduce the risk of BPD or to improve its outcome may be informed by a more complete understanding of the molecular mechanisms involved in the pathogenesis of BPD (Cerny et al. 2008).

Genetic predisposition may play a major role in the development of BPD (Bhandari and Gruen 2006; Lavoie and Dube 2010). Genetic association studies relating to BPD have primarily focused on components of the innate immune and antioxidant defense systems, mechanisms of vascular and lung remodeling and surfactant proteins (Lavoie and Dube 2010). Although studies to date have not been able to identify reproducible genetic risk markers for BPD, they have directed us toward new research avenues. The nuclear factor I (*Nfi)* gene family encodes site‐specific transcription factors essential for the development of a number of organ systems throughout development (Nagata et al. 1983; de Jong and van der Vliet 1999). The *Nfi* gene family contains four different but highly related genes: *Nfia, Nifb, Nfic,* and *Nfix* (Kruse and Sippel 1994; Chaudhry et al. 1998). Binding sites for these factors are identified in promotor, enhancer, or silencer regions for a plethora of genes in almost every organ and tissue (Kruse and Sippel 1994; Gronostajski 2000). *Nfib* is commonly expressed throughout the human body regulating cell differentiation and proliferation (GTEx Consortium, 2015; Uhlen et al. 2015). When overexpressed, *Nfib* regulates both cell viability and cell proliferation in murine small cell lung cancer transformation (Dooley et al. 2011) and it promotes metastasis through widespread increase in chromatin accessibility (Denny et al. 2016), suggesting it plays a critical role in cancer development (Becker‐Santos et al. 2017). However, it exhibits tumor‐suppressive functions in various other malignancies suggesting it may have diverse functions in many cell types (Becker‐Santos et al. 2017).

*Nfib* is essential for late gestational lung maturation (Steele‐Perkins et al. 2005), and *Nfib*‐deficient (−/−) animals show defects in lung development and die at birth with immature lungs (Grunder et al. 2002; Steele‐Perkins et al. 2005). Lung maturity is delayed in both *Nfib* (−/+) hemizygous and homozygous (−/−) mice, but to a different extent (Grunder et al. 2002; Steele‐Perkins et al. 2005). *Nfib* null mice die within 24 h of birth, and 60% of *Nfib* hemizygous newborns survive past 24 h of age (Grunder et al. 2002). This suggests that loss of the *Nfib* gene (either one or both alleles) leads to delay in lung maturation and reduced survival after birth, and the hemizygous survivors offer an opportunity for further study.

Lung development is normal in *Nfib* null mice at E15.5 with severely delayed lung maturation at E17.5 (Steele‐Perkins et al. 2005) and arrested lung development in the late pseudoglandular or early canalicular stage at E18.5 (Grunder et al. 2002; Steele‐Perkins et al. 2005). An increase in the total DNA content‐to‐lung mass ratio in the *Nfib* −/− mice compared to the wild‐type mice may suggest a defect in apoptosis or cell proliferation required for lung maturation (Steele‐Perkins et al. 2005). Proliferating cell nuclear antigen (PCNA) and fibroblast growth factor‐10 mRNA were increased, while surfactant protein mRNAs (type II cell marker) and *Aquaporin‐1* and epithelial sodium channel gene (*ENaC*) (type I cell markers) were decreased in *Nfib* −/− mice, confirming that *Nfib* is necessary for lung development (Steele‐Perkins et al. 2005). Lungs of *Nfib* hemizygous mice demonstrate definite histologic evidence of delayed lung development in the late canalicular stage at E18.5, with only focal transitions to the terminal sac stage (Grunder et al. 2002) with a decrease in type I and type II cell differentiation (Steele‐Perkins et al. 2005). The loss of the *Nfib* gene (either one or both alleles) leads to delay in lung maturation and reduced survival after birth. The effects of oxygen in a genetically predisposed lung with delayed lung maturation have not been well studied. The relative resistance to the toxic effects of oxygen on the lung may depend not only on the duration of O_2_ exposure in neonatal and adult animals but also on the ability of the animal to mount an antioxidant enzyme response (Frank et al. 1978). Adult animal species such as rodents, are much more susceptible to the toxic effects of oxygen compared to human newborns (Frank et al. 1978). We hypothesized that the *Nfib* hemizygous (heterozygous) adult mice would have a lower survival in hyperoxia compared to adult wild‐type littermates.

## Materials and Methods

The C57BL/6 *Nfib* hemizygous (Hz) mouse and wild‐type littermate mice were bred as per protocol (Steele‐Perkins et al. 2005) and reared at the Laboratory Animal Facility (LAF), Roswell Park Cancer Institute, Buffalo, NY. With IACUC approval, adult mice were transferred to LAF, University at Buffalo at 11–13 weeks of age for hyperoxia exposure studies.

### Survival studies

Following acclimatization for 3 days, both wild‐type (Wt) and hemizygous (Hz) littermates were exposed to 100% O_2_ in a Plexiglass chamber, monitored for temperature, oxygen concentration, and humidity (50–60%). During O_2_ exposures mice were monitored frequently for signs of distress (breathing, activity, pilo erection, and cyanosis) and any animal deemed to be in distress was sacrificed. With the death of *Nfib* hemizygous mice after 83 h of O_2_ exposure and wild‐type mice dying at earlier time points, survival studies were repeated following exposure to 100% O_2_ for 80, 72, and 66 h in both mice groups. With no deaths at 66 h of O_2_ exposure, this time point was selected for additional study outcomes in both groups.

A total of 46 mice were exposed to hyperoxia (19 *Nfib* Hz mice and 27 Wt mice) for survival studies; additional hemizygous mice and wild‐type littermates were exposed to hyperoxia or to room air for 66 h for comparison. Mice in all groups (*Nfib* Hz with and without O_2_; Wt with and without O_2_) were euthanized by intraperitoneal injection of sodium pentobarbital. The right lung was formalin fixed for histopathology and the left lung was flash frozen in liquid nitrogen for RNA extraction and PCR analysis (*N* = 5 in each group). Frozen lung tissue was stored at −80°C until processed.

### Bronchoalveolar lavage

Bronchoalveolar lavage (BAL) was collected in *Nfib* hemizygous and Wt mice following 66 h of exposure to O_2_ or room air (*N* = 4 in each group). The trachea was cannulated and the airways were instilled with normal saline (1 mL × 3). Approximately 0.8 mL of saline was recovered per wash. The lavage fluid was centrifuged at 150 *g* for 10 min at 4°C. The supernatant of the first aliquot was stored at −80°C for cytokine assays. The pellet collected from the centrifuged tube was used for the cell counts. Cell counts were performed on the Coulter Counter Multisizer 3. Differential count was performed by counting cells on cytospin preparations of the resuspended cell pellet stained with diff‐Quik stain (Fisher Scientific, Pittsburgh, PA).

### Albumin and cytokine measurements

The albumin levels in the BAL supernatants were measured by mice albumin ELISA Quantitation assay (Bethyl laboratories Inc, Montgomery, TX). Cytokines were measured by Luminex‐100 multiplex cytokine assay. The cytokines measured included interleukin‐1*β* (Il‐1*β*), interleukin‐6 (IL‐6), monocyte chemotactic protein 1 (MCP‐1), macrophage inflammatory protein 2 (MIP‐2), and keratinocyte chemoattractant (KC) protein.

### RNA isolation

Lung tissue was weighed and ground to fine powder using a mortar and pestle under liquid nitrogen. The tissue was then homogenized in appropriate buffer for isolation of RNA using RNeasy Mini kit (Qiagen, Valencia, CA) with on column DNAase digestion per the manufacturer's protocol. RNA integrity was assessed using Experion Automated Electrophoresis System (BioRad, Hercules, CA).

### Whole lung gene expression profiling by RT^2^ qPCR

The PCR array performs gene expression analysis with real‐time PCR sensitivity and the multigene profiling capability of a microarray. Pathway focused gene expression profiling for angiogenesis (PCR Array: PAMM‐024) and for oxidative stress (PCR Array: PAMM‐065) was performed using RT^2^Profiler PCR Array (SA Biosciences, Frederick, MD). The angiogenesis and the oxidative stress PCR array profiles the expression of 84 specific genes that regulate angiogenesis and oxidative stress, respectively. Using RT^2^ first strand kit (SA Biosciences, MD), 500 ng of total RNA was reverse transcribed to cDNA, which was mixed with RT^2^‐SYBR Green qPCR master mix. Aliquots of this mix were placed into each of the PCR array plates containing the predispensed gene‐specific primer sets and PCR performed on a 96‐well MyiQ thermocycler (BioRad, Herculus, CA) according to the manufacturer's protocol. The instrument's software was used to calculate the threshold cycle (C_t_) values for all genes on the PCR Array. Fold change in gene expression for pair‐wise comparison was processed on the excel‐based PCR Array Data Analysis software (SA Biosciences) using the equation 2^−∆∆Ct^ by comparing the control group (WT‐RA) to the other three groups (WT‐O_2_; *Nfib*‐O_2_; and *Nfib*‐RA).

### Histopathology

The trachea was cannulated and lungs instilled with 10% buffered formalin at 25 cm of H_2_O pressure. The lungs were fixed overnight, serially dehydrated in ethanol and embedded in paraffin. To evaluate the general lung architecture, embedded lungs were cut into 5‐μm‐thick sections and stained with hematoxylin and eosin. We analyzed five sections/mouse and at least 20 fields/section. Assessment of alveolarization was done by measuring radial alveolar count (Emery and Mithal 1960).

### Statistical analysis

All data are expressed as mean ± standard deviation (SD). *P* values for gene expression were calculated by student's test of the replicate of 2^∆∆Ct^ value for each gene in the control group and the treatment group (Rao et al. 2013). Fisher Exact test, unpaired t‐test, and ANOVA were used to compare differences among groups. A *P* value of <0.05 was considered significant.

## Results

### Survival data

**Figure 1:**
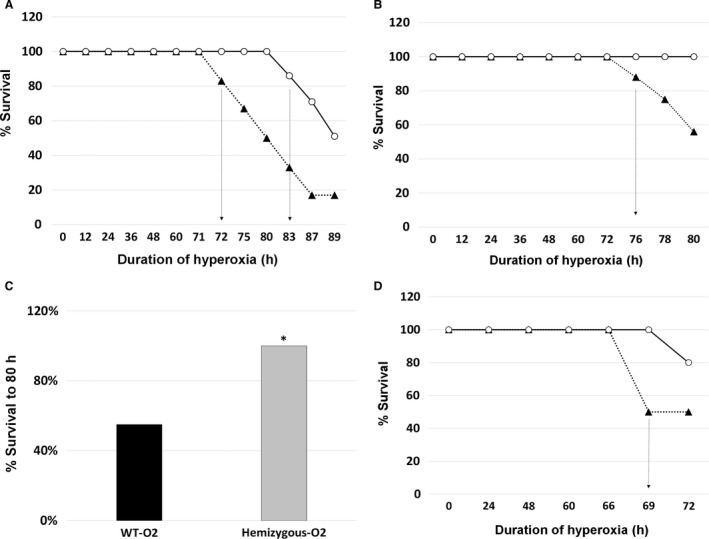
Survival studies. *Nfib* hemizygous mice (white circles) and wild‐type littermates (black triangles) were exposed to 89 h (A); 80 h (B) or 72 h (D) of 100% O_2_. Wild‐type mice died at earlier time points in all three experiments. The LD_50_ for Wt‐O_2_ group was 80 h versus 89 h for the Hz‐O_2_ group (A). Survival was significantly higher in *nfib* hemizygous mice after 80 h of hyperoxia (*P *<* *0.05 vs. Wt‐O_2_ group; Fisher's Exact Test; C). Arrows point to first death in the three experiments in each group.

The first exposure was to evaluate the survival time of the hemizygous mice in hyperoxia compared to their wild‐type littermates. Contrary to our expectations, the hemizygous mice survived longer than the wild‐type mice, with the first wild‐type mouse dying at about 72 h of exposure and the first hemizygous mouse dying at 83 h (Fig. 1A). The LD_50_ was 80 h in the WT group versus 89 h in the Hz group, respectively. The survival rate to 89 h for wild‐type littermates was 17% (1/7) compared to 51% (4/7) in hemizygous mice.

Survival experiments were repeated to confirm the reproducibility of the results in both groups (Fig. 1B). The second hyperoxia exposures were limited to 80 h and the LD_50_ was 80 h in the Wt group. The survival rate after 80 h of hyperoxia exposure for wild‐type littermates was 56% (9/16) compared to 100% (7/7) for the hemizygous mice (Fig. 1B). Combining the survival data of the above two hyperoxia exposures, hemizygous mice had a significantly higher survival rate (100% – 14/14) in hyperoxia compared to the wild‐type littermates (55% – 12/22) at 80 h of exposure (*P* < 0.01; Fisher Exact Test; Fig. 1C).

The third set of hyperoxia exposure experiments was set for 72 h so we could better study the response of *Nfib* hemizygous mice compared to wild type at a moderately high level of lung injury, but not an extreme level immediately prior to death. Similar to previous exposures, wild‐type mice died earlier, with an LD_50_ of 72 h. The survival rate at 72 h was 50% (2/4) in the Wt group versus 80% (4/5) in the Hz group (Fig. 1D). These experiments were done to establish the final time point for hyperoxia exposure for outcome studies, which was set at 66 h. There were no deaths at this time point in either of the groups. All data below are at the 66 h hyperoxia exposure time.

### BAL cell counts and albumin

**Figure 2:**
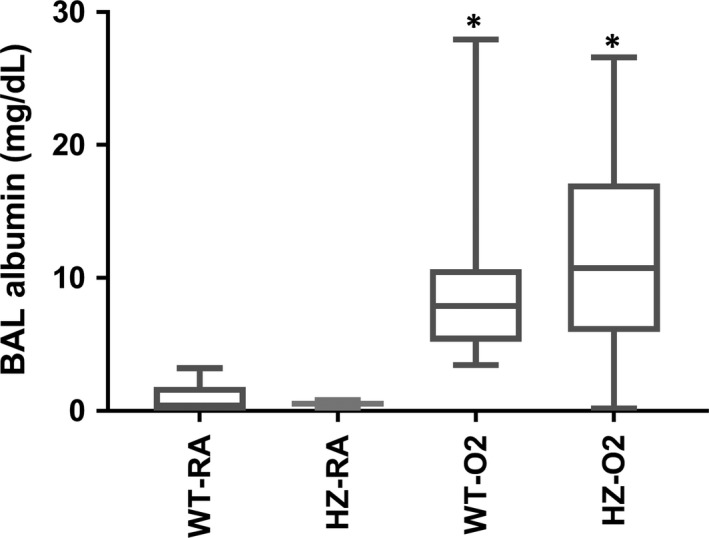
Bronchoalveolar lavage (BAL) albumin levels. There was no significant difference in albumin levels either between the two room air groups (wild‐type room air [Wt‐RA] vs. hemizygous room air) (Hz‐RA) groups; or the hyperoxia groups (wild‐type hyperoxia [Wt‐O_2_] vs. hemizygous hyperoxia [Hz‐O_2_] groups). A significantly higher albumin levels was noted in the BAL in the hyperoxia groups vs. corresponding RA groups (*P < 0.01 vs. corresponding RA group, unpaired *t*‐test).

There was no difference in BAL total cell counts or differential count between wild‐type and hemizygous mice when exposed to either hyperoxia or room air (Table 1). There was no significant difference in BAL albumin between wild‐type and hemizygous mice exposed to hyperoxia (10.1 ± 8.2 mg/mL vs. 11.7 ± 8.6 mg/mL, respectively) (Fig. 2); similarly, the albumin levels were not different between the two room air groups (0.84 ± 1.3 mg/mL vs. 0.5 ± 0.3 mg/mL, respectively) (Fig. 2). As expected, BAL albumin levels were significantly higher in the hyperoxia groups compared to corresponding room air groups (*P* < 0.05, ANOVA) (Fig. 2).

**Table 1:** Bronchoalveolar lavage analysis in wild‐type and hemizygous mice

Bronchoalveolar Lavage	Wt–RA	Hz–RA	Wt–O_2_	Hz–O_2_
Total count (10^3^ cells/mL)	110.8 ± 45.6	151.7 ± 131	156.8 ± 78.5	159 ± 45
Differential count				
Macrophages	100	100	80	93
Neutrophils	0	0	16	4
Lymphocytes	0	0	4	3
Cytokines in BAL (pg/mL)				
Interleukin 6 (IL‐6)	ND	ND	25.3 ± 8.8	24.4 ± 9.7
Keratinocyte chemoattractant (KC)	ND	ND	23.1 ± 14	39.6 ± 16.2
Macrophage chemoattractant protein 1 (MCP‐1)	ND	ND	11.1 ± 6.0	22.9 ± 4.2*
Macrophage inhibitory protein 2 (MIP‐2)	ND	ND	70 ± 16	30 ± 3*

Values expressed as mean ± SD; ND – not detectable (below assay detectable levels); *P < 0.05 vs. Wildtype‐O_2_. WT – RA, wild‐type room air; HZ‐RA, hemizygous room air; WT‐O_2_, wild‐type hyperoxia; HZ‐O_2_, hemizygous hyperoxia group.

### BAL cytokines

IL‐6, KC, MCP‐1, and MIP‐2 were expressed below detectable levels in the lavage fluid in both the wild‐type and hemizygous mice exposed to room air. Among the four cytokines, interleukin‐6 (IL‐6) and keratinocyte chemoattractant (KC) were not significantly different between the two hyperoxia groups (Table 1). Macrophage inhibitory protein 2 (MIP‐2) was significantly higher in the wild‐type mice exposed to hyperoxia; however, macrophage chemoattractant protein 1 (MCP‐1) was significantly higher in the lavage fluid in hemizygous mice exposed to hyperoxia (Table 1).

### Lung histology

**Figure 3:**
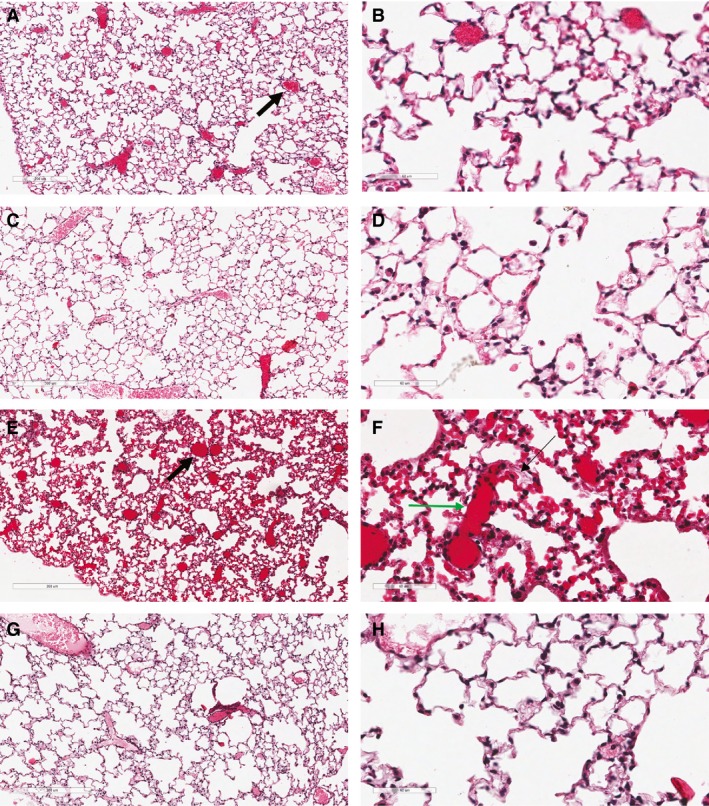
Lung histopathology of wild‐type (A, B, E, and F) and hemizygous (C, D, G, and H) mice exposed to 72 h (A–D) or 80 h (E–H) of hyperoxia (100×‐A, C, E, and G (left panel of diagram); 400×‐B,D,F,H (right panel of diagram). Wild‐type mice demonstrate increased vascularity with new vessel formation at 72 and 80 h (thick black arrow‐A/E) with pulmonary hemorrhages (green arrow‐F) and alveolar edema at 80 h (thin black arrow‐F) (E and F). Hemizygous mice had relatively normal lung architecture and no increased vessel density at 72 and 80 h of O_2_ exposure (72 h – C, D; 80 h – G, H).

**Figure 4:**
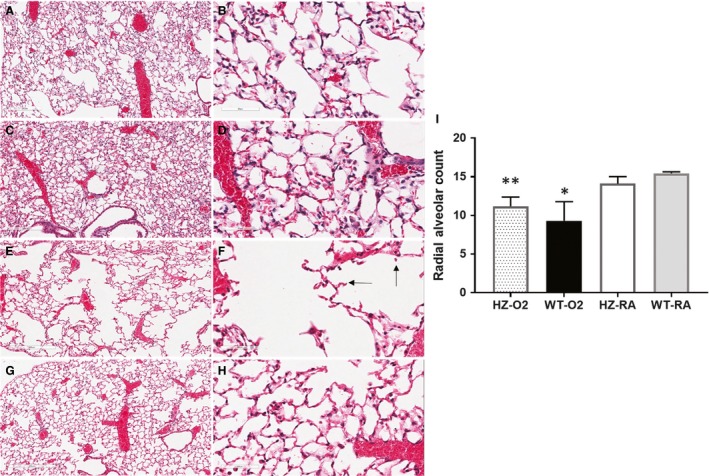
Lung histopathology of wild‐type (A, B, E, and F) and hemizygous (C, D, G, and H) mice exposed to room air (A–D) or hyperoxia (E–H) of hyperoxia; 100×‐A, C, E and G (left panel of diagram); 400×‐B, D, F and H (right panel of diagram). After 66 h of O_2_ exposure, wild‐type mice demonstrate enlarged alveoli (E and F) with loss of secondary septae (black arrows‐F); with normal lung histology in hemizygous mice exposed to hyperoxia (G and H). Hemizygous mice and wild‐type mice exposed to room air had relatively normal lung architecture (A–D). Alveolar simplification was quantified by radial alveolar count (RAC). Wt‐O_2_ group had significantly lower RAC compared to the other three groups (*P < 0.001 vs. HzO_2_, HzRA, and WTRA groups; Fisher's post hoc test, ANOVA). Hz‐O_2_ group had significantly lower RAC compared to room air groups (*P < 0.001 vs. HzRA and WTRA groups; Fisher's post hoc test, ANOVA).

With decreased survival of wild‐type mice in hyperoxia at 72 h and at 80 h compared to hemizygous mice, we studied the histopathology of the lungs in these groups at both time points (Fig. 3). Hemizygous mice exposed to hyperoxia demonstrated an essentially normal alveolar architecture, airway pattern, and vessel distribution at 72 h and at 80 h (72 h: Fig. 3C and D; 80 h: 3G and H). This is in contrast to an increase in new vessel formation (72 h: Fig. 3A and B; 80 h: 3E and F) and pulmonary hemorrhages seen in wild‐type mice at 80 h (Fig. 3E and F). Lungs exhibited edema, congestion, and intraalveolar hemorrhage with areas of alveolar collapse particularly at 80 h (Fig. 3E and F). We studied lung histopathology at 66 h in all the four groups. Both of the room air groups (wild type and hemizygous) had well‐preserved alveolar histology on lung sections (WT: Fig. 4A and B; Hz: Fig. 4C and D). In comparing the two hyperoxia groups, the lungs of wild‐type mice exhibited areas of alveolar enlargement and destruction, blunting of secondary septae, and intraalveolar hemorrhage (Fig. 3F, 4E). Hemizygous mice exposed to hyperoxia had a relatively normal alveolar histology compared to the wild‐type group (Fig. 3H, 4G). Hyperoxia groups had significantly lower radial alveolar count compared to room air groups (Fig. 3I, *P < 0.001 vs. HzRA and WTRA groups; Fisher's post hoc test, ANOVA). However, wild‐type mice had a significantly lower radial alveolar count compared to hemizygous mice exposed to hyperoxia (Fig. 3I; *P < 0.001 vs. HzO_2_ group; Fisher's post hoc test, ANOVA). Vascular congestion, intraalveolar hemorrhage and edema may have contributed to respiratory failure and death secondary to oxygen toxicity at 80 h in wild‐type mice.

### Angiogenic gene expression

To further address the hyperoxia resistance of the hemizygous mice we analyzed 84 angiogenic genes by real‐time PCR array analysis. Connective tissue growth factor (*Ctgf*) was overexpressed in both the wild‐type (Wt‐O_2_) and hemizygous mice (HZ‐O_2_) exposed to O_2_ (Table 2) compared to the corresponding room air group. However, the hypoxia‐inducible factor 1*α* (*Hif1a*) and thrombospondin 1 genes were upregulated only in the HZ‐O_2_ group. Colony‐stimulating factor 3 (*Csf3*), sphingosine kinase 3 (*Sphk1*), and transforming growth factor *α* (*Tgfa*) genes were significantly downregulated in hemizygous (HZ‐O_2_) versus wild‐type mice exposed to hyperoxia.

**Table 2:** Real‐time PCR array analysis of 84 angiogenic genes was analyzed in lung homogenate of wild‐type (nfib +/+) (Wt) and hemizygous (nfib +/−) (Hz) adult mice exposed to 66 h of 100% O_2_ or room air. The following gene expression comparisons were performed: Wt‐RA versus Wt‐O_2_; Hz‐RA versus Hz‐O_2_; Wt‐RA versus Hz‐RA; Wt‐O_2_ versus Hz‐O_2_. Genes are expressed as the fold change in expression of the same gene in the control group (Gene expression in the control group = 1.0). Genes that are significantly expressed (*P* < 0.05) are presented in the table (*n* = 5 mice in each group)

Overexpressed genes	Fold change	Underexpressed genes	Fold change
Gene expression in Wt‐O_2_ group (Control – Wt‐RA group)			
*Ctgf* – connective tissue growth factor	7.7		
*Sphk1* – sphingosine kinase 1	7.4		
Gene expression in Hz‐O_2_ group (Control – Hz‐RA group)			
*Ctgf* – connective tissue growth factor	4.7	*Ccl1* – chemokine ligand 1	−6.5
*HIF‐1α* – hypoxia‐inducible factor 1*α*	10.2		
*Thbs1* – thrombospondin 1	6.6		
Gene expression in Hz‐O_2_ group (Control – Wt‐O_2_ group)			
		*Csf3* – colony‐stimulating factor 3	−2.8
		*Spkh1* – sphingosine kinase 1	−2.3
		*Tgfα* – transforming growth factor alpha	−2.4

### Oxidative stress gene expression

Wild‐type mice overexpressed some of the enzymes involved in redox regulation upon exposure to hyperoxia compared to the room air group (Table 3). Glutathione peroxidase 2; NAD dehydrogenase quinone 1; prostaglandin endoperoxide synthase 2; sulfiredoxin and theoredoxin reductase 1 were significantly upregulated in wild‐type mice exposed to supplemental oxygen. This is in contrast to significant downregulation of several genes involved in redox signaling seen in hemizygous mice following exposure to hyperoxia; 13 genes were significantly downregulated (Table 3) in hemizygous mice and no genes were overexpressed in this group. There were no significant differences in gene expression between the two hyperoxia or room air groups among wild‐type and hemizygous mice.

**Table 3:** Real‐time PCR array analysis of 84 genes involved in oxidative stress was analyzed in lung homogenate of wild‐type (nfib +/+) (Wt) and hemizygous (nfib +/−) (Hz) adult mice exposed to 66 h of 100% O_2_ or room air. The following gene expression comparisons were performed: Wt‐RA versus Wt‐O_2_; Hz‐RA versus Hz‐O_2_; Wt‐RA versus Hz‐RA; Wt‐O_2_ versus Hz‐O_2_. Genes are expressed as the fold change in expression of the same gene in the control group (gene expression in the control group = 1.0). Genes that are significantly expressed (*P* < 0.05) are presented in the table (*n* = 5 mice in each group)

Overexpressed genes	Fold Change	Underexpressed genes	Fold change
Gene expression in Wt‐O_2_ group (Control – Wt‐RA group)			
*Gpx2* – glutathione peroxidase 2	8.0	*Noxo1* – NADPH oxidase organizer 1	−4.3
*Nqo1* – NAD dehydrogenase, quinone 1	4.8	*Nudt15* – Nudix hydroxylase 15	−4.3
*Ptgs2* – prostaglandin endoperoxide synthase 2	4.2		
*Srxn1* – sulfiredoxin 1	8.7		
*Txnrd1* – thioredoxin reductase 1	8.4		
Gene expression in Hz‐O_2_ group (Control – Hz‐RA group)			
		*Ccs* – Copper chaperone for SOD	−4.8
None were significant		*Duox1* – Dual Oxidase 1	−4.9
		*Epx* – Eosinophil peroxidase	−4.9
		*Gpx5/6/7* – Glutathione peroxidase 5/6/7	−4.9
		*IL19/22* – Interleukin 19/22	−4.9
		*LPO* – Lactoperoxidase	−4.9
		*MPO* – Myeloperoxidase	−4.8
		*Noxo1* – NADPH oxidase organizer 1	−4.9
		*Rag2* – Recombination activating gene 2	−4.8
		*Noxa1* – NADPH oxidase activator 1	−4.9

## Discussion

Transcription factor, *Nfib* plays a fundamental role in various biologic process, particularly in developmental regulation of cell differentiation in a number of organ systems (Becker‐Santos et al. 2017). *Nfib*‐deficient mice possess unique defects in lung maturation; *Nfib* null mice develop severely delayed lung development and die at birth (Grunder et al. 2002) with *Nfib* hemizygous mice surviving beyond 24 h of birth (Grunder et al. 2002; Steele‐Perkins et al. 2005). These findings suggest that *Nfib* may be a critical transcription factor for fetal lung maturation. It is conceivable that *Nfib* hemizygous mice would be susceptible to acute lung injury and death from hyperoxia. Interestingly, our experiments demonstrated that adult *Nfib* hemizygous mice had better survival compared to wild‐type littermates following exposure to hyperoxia.

Adult animals from various species such as rats, mice, and rabbits succumb to O_2_‐induced lung toxicity within 3–5 days (Frank et al. 1978). However, immature rats demonstrate the capacity to increase antioxidant enzyme (AOE) activity and are relatively resistant to hyperoxic exposure, whereas adult rats are deficient in their lung antioxidant enzyme (AOE) responsiveness and are more susceptible to toxic effects of an increase in oxygen‐free radicals (Frank 1991). We hypothesized that the *Nfib* hemizygous mice would be more susceptible to the toxic effects of O_2_ compared to their wild‐type littermates; however, wild‐type mice died at a higher rate compared to hemizygous mice at ≥66 h of O_2_ exposure. The decreased survival of hyperoxia‐exposed wild‐type mice may be related to overwhelming free radical damage and systemic inflammatory response as reported in the literature (Frank 1991). The pathological changes in edema, vascular congestion, and prominent intraalveolar hemorrhage noted in wild‐type mice at both 72 and 80 h are characteristic of pulmonary oxygen toxicity (Clark and Lambertsen 1971). Pulmonary edema with widening of interstitial spaces, a prominent pathologic finding of oxygen toxicity, is also demonstrated in these mice. The prominence of these changes is dependent on the age of the experimental animal, as immature mice are more resistant than older mice (Clark and Lambertsen 1971). However, none of these changes is noted in hemizygous mice despite similar hyperoxia exposure. Assessment of bronchoalveolar lavage and selected cytokines may suggest an inflammatory response of greater degree in the wild‐type mice compared to hemizygous group when exposed to hyperoxia. Macrophage inhibitory protein (MIP‐2) was higher in the lavage fluid of wild‐type mice; however, macrophage chemoattractant protein 1 (MCP‐1) was higher in the hemizygous group when exposed to oxygen. The cytokine, MIP‐2 produced by monocytes, macrophages, and dendritic cells, stimulate neutrophils to secrete inflammatory cytokines such as interleukin‐6 and interleukin‐1 (Deng et al. 2000; De Filippo et al. 2008). Even though neutrophils were higher in lavage fluid of Wt‐O_2_, this was not significantly different from the Hz‐O_2_ group. Albumin in lavage fluid was higher in both the groups exposed to hyperoxia, implying an inflammatory response to a greater or lesser extent with acute lung injury. Free radicals promoting synthesis of inflammatory mediators both directly and indirectly via nuclear transcription factors, especially in the presence of hyperoxia may explain lung injury and mortality in Wt‐O_2_ group (Welty et al. 1993; Closa and Folch‐Puy 2004).

*Nfib* is expressed in both mesenchyme and epithelium during lung development; however, loss of *Nfib* specifically in mesenchyme affects both mesenchyme and epithelial cell proliferation and distal epithelial cell differentiation (Hsu et al. 2011). Loss of both alleles for *Nfib* is lethal; survival to adulthood is possible in the hemizygous state, suggesting hemizygous mice may have developed cellular mechanisms for survival. The absence of pathological changes in oxygen toxicity indicates relative resistance to administered oxygen and hence enhanced survival in hemizygous mice. Identification of factors responsible for this resistance could contribute to a better understanding of the mechanisms of pulmonary oxygen toxicity. Nuclear factor I/B (Nfib) is a transcription factor required for proper development and regulation of cell differentiation in several tissues including the brain and the lung (Gronostajski 2000; Grunder et al. 2002). Hyperoxia may elicit an impaired hypophyseal–adrenocortical stress response in hemizygous mice; as oxygen tolerance is associated with impaired stress response (Clark and Lambertsen 1971). Lack of cell differentiation in hemizygous mice, may lower cellular metabolism increasing oxygen tolerance, as enzymes and metabolic pathways are susceptible to toxic effects of oxygen (Clark and Lambertsen 1971).

MicroRNAs (miRNAs) control gene expression by translational inhibition and destabilization of mRNAs and are implicated in tissue morphogenesis, cellular processes like apoptosis and major signaling pathways (Kloosterman and Plasterk 2006). MicroRNAs that control balance of signals during fetal lung development are likely silenced in adult lung cells but reactivate to create a developmental‐like cell phenotype during stress such as tumor initiation and progression (Becker‐Santos et al. 2016). *Nfib* that is essential for fetal lung maturation is the major target of oncofetal miRNAs during development (Becker‐Santos et al. 2016). The same oncofetal miRNAs were upregulated with associated downregulation of *Nfib* in adults with non‐small cell lung cancer (Becker‐Santos et al. 2016), implying a functional role for *Nfib* in regulating cell differentiation. This also suggests the commonalities between processes regulating normal development and tumorigenesis. The fetus is uniquely positioned to protect itself from the harmful effects of hypoxia and hyperoxia, as its growth is driven by hypoxia‐inducible factor (HIF). HIF‐1*α* and thrombospondin‐1 genes are overexpressed in adult hemizygous mice exposed to supplemental O_2_. We contemplate whether fetal miRNAs may target *Nfib* and HIF transcription to regulate cell proliferation and fetal survival. We speculate whether hemizygous mice revert to fetal phenotype develop when exposed to oxygen as adults contributing to relative resistance to hyperoxia. Overexpression of HIF‐1*α* might have contributed to increased survival in these mice; HIF‐1*α* overexpression has been related to progression free and overall survival in certain human cancers (Bhaskar and Tiwary 2016).

This is the first study to demonstrate the protective effect of *Nfib* hemizygous mice from hyperoxia‐induced mortality compared to wild‐type mice. Microarray data demonstrated a downregulation of the colony‐stimulating factor 3 (C*cf3*) gene that controls the production, differentiation and function of neutrophils and monocytes/macrophages, may explain the lack of inflammation in hemizygous mice. The role of miRNAs in posttranscriptional silencing of MCP‐1 is well studied (Nakamachi et al. 2009), and may contribute to better survival in hemizygous mice. Overexpression of some of the redox genes, including those involved in expression of AOE activity is typical following exposure to hyperoxia. It is unusual for downregulation of genes involved in oxidative stress signaling in the presence of hyperoxia, as seen in hemizygous mice. We can only speculate whether downregulation of genes involved in redox signaling along with the associated lack of inflammation; results in relatively normal lung histology contributing to prolonged survival of hemizygous mice.

*Nfib* hemizygous mice possess unique defects in lung maturation as 40% of these mice die within 24 h of birth. It is essential to examine the lungs in mice that die within 24 h and how they differ compared to their siblings that survive past the 24‐h period into adulthood. This study comes with significant limitations. The study was underpowered for analysis of inflammation in lavage fluid due to small numbers of mice studied in each group. We did not measure antioxidant enzyme activity as part of this experimental protocol. The PCR array provides data on gene expression changes but important functional changes from protein expression induced by posttranslational modifications were not identified. The gene expression changes are representative of the whole lung, although individual cell types may have a specific pattern of susceptibility to oxidant injury and inflammation. As *Nfib* is the putative target of oncofetal miRNAs, it is essential to elucidate the potential functional role of *Nfib* and its miRNA regulators in development and disease, particularly with reference to hyperoxia exposure. Despite these limitations, we report for the first time that *Nfib* hemizygous adult mice are relatively more resistant to hyperoxia‐induced lung injury compared to wild‐type mice. However, the mechanisms responsible for this resistance still need clarification. Insights into mechanisms of hyperoxia resistance could have huge implications especially in the context of lung immaturity and bronchopulmonary dysplasia. This may open novel avenues of lung protective strategies at birth.

## Conflict of Interest

None declared.
